# Computational Docking of Antibody-Antigen Complexes, Opportunities and Pitfalls Illustrated by Influenza Hemagglutinin

**DOI:** 10.3390/ijms12010226

**Published:** 2011-01-05

**Authors:** Mattia Pedotti, Luca Simonelli, Elsa Livoti, Luca Varani

**Affiliations:** Institute for Research in Biomedicine, via Vela 6, 6500 Bellinzona, Switzerland; E-Mails: mattia.pedotti@irb.unisi.ch (M.P.); luca.simonelli@irb.unisi.ch (L.S.); elsa.livoti@irb.unisi.ch (E.L.)

**Keywords:** antibody modeling, computational docking, influenza, hemagglutinin, antibody-antigen complexes

## Abstract

Antibodies play an increasingly important role in both basic research and the pharmaceutical industry. Since their efficiency depends, in ultimate analysis, on their atomic interactions with an antigen, studying such interactions is important to understand how they function and, in the long run, to design new molecules with desired properties. Computational docking, the process of predicting the conformation of a complex from its separated components, is emerging as a fast and affordable technique for the structural characterization of antibody-antigen complexes. In this manuscript, we first describe the different computational strategies for the modeling of antibodies and docking of their complexes, and then predict the binding of two antibodies to the stalk region of influenza hemagglutinin, an important pharmaceutical target. The purpose is two-fold: on a general note, we want to illustrate the advantages and pitfalls of computational docking with a practical example, using different approaches and comparing the results to known experimental structures. On a more specific note, we want to assess if docking can be successful in characterizing the binding to the same influenza epitope of other antibodies with unknown structure, which has practical relevance for pharmaceutical and biological research. The paper clearly shows that some of the computational docking predictions can be very accurate, but the algorithm often fails to discriminate them from inaccurate solutions. It is of paramount importance, therefore, to use rapidly obtained experimental data to validate the computational results.

## 1. Introduction

Individuals that recover from the attack of a pathogen have antibodies (Abs) capable of detecting and neutralizing the same pathogen in a future encounter, usually conferring life-long protection from it. Detection and neutralization are initiated by the binding of these antibodies to antigens, often surface proteins, through specific atomic interactions between the antibody and the region of the antigen (Ag) that it recognizes (epitope). A better understanding of these interactions is expected to accelerate vaccine development, since most current vaccines are based on the generation of neutralizing antibody responses. If we understand the structural rules governing Ab-Ag interactions in a given virus, for instance, then we have the molecular basis to attempt to design and synthesize new epitopes to be used as vaccines, optimize the antibodies themselves for passive immunization or design new drugs mimicking the antibodies or their effect.

In addition to pharmaceutical development, antibodies play an increasingly relevant role in basic research and industrial processes, where they are starting to be used as recognition elements sensitive to the presence of a given antigen. Designing and synthesizing new antibodies with desired properties would, therefore, have a profound impact, but we are very far away from being able to do that. Despite antibodies having been known and characterized for several decades [1,2], in fact, we still know remarkably little about their interactions. Given an antibody structure, for instance, we cannot even predict whether it can bind a protein, nucleic acid or sugar, let alone the specific antigen or conformational epitope that it recognizes. The study of Ab-Ag complexes should further our understanding of the general principles of recognition and, in the long run, gives us the basis for the successful design of new molecules or the rational optimization of existing ones.

The best way to study atomic interaction is to obtain the three-dimensional structure of antibody-antigen complexes. Traditionally, this is achieved by experimental techniques like X-ray crystallography, an often long and laborious process with high failure rate. Thanks to advances in algorithms and processing power, however, we can now use computational techniques for the structural characterization of intermolecular complexes. Computational docking—the process of predicting the conformation of a complex starting from its separated components—provides a fast and inexpensive route to obtain structures, including those which are not suitable for experimental determination. Although computational docking is still in its infancy and marred by several limitations, there is no doubt that it will become more and more accurate, relevant and widespread in the coming years.

Here we first illustrate the application of computational docking to the study of antibody-antigen interactions, and then highlight the strengths and weaknesses of the approach by predicting the binding of two different antibodies to hemagglutinin, the surface protein of influenza virus and an important pharmaceutical target. Being able to accurately predict those structures, for which X-ray information is available, would strengthen our belief that computational techniques can be used to characterize the binding of new antibodies against the same epitope.

### 1.1. Computational Docking

Computational docking, a relatively new and constantly evolving technique, is the process of predicting the structure of a multi-molecular complex from the structures of its separated components. Its progress has been monitored since 2002 by the “Critical Assessment of PRediction of Interactions” project (CAPRI) [3], a comparative evaluation of protein-protein docking algorithms on a set of known targets. Here we focus on docking of antibodies to protein antigens, which presents specific challenges but also has peculiar features exploitable to ease the calculations.

In a typical docking protocol, the structures of the antigen and antibody are separated by approximately 25 Å and subsequently brought together by the chosen algorithm. The first necessary step, therefore, is obtaining the structures of the isolated antigen and antibody. The starting structure may be defined as follows:

- “Bound”, if it originates from an experimental structure of the complex that needs to be docked. This is interesting when developing docking procedures but it is generally not biologically attractive, because computational docking is unlikely to add relevant information if an experimental structure is already available.
- “Unbound”, if it originates from an experimental structure of the molecule not bound to the partner that needs to be docked, *i.e.*, either free or bound to a different partner. This is the most common scenario for antigens, especially since the number of available protein structures is increasing thanks to several structural genomics efforts. Structures of free antibodies, instead, are usually not available, nor they would be particularly useful since Abs are known to drastically change conformation upon binding [4].
- “Modeled”, if it has been predicted by homology modeling and/or other computational techniques like *ab initio* predictions or molecular dynamics. A thorough description of homology modeling for protein antigens is beyond the scope of this manuscript. Suffice to say that the results are remarkably accurate if the target protein has sequence similarity to a protein with known structure and that even *ab initio* predictions are starting to produce accurate results, albeit much less than homology modeling [5–7]. Antibody structures can be predicted with remarkable accuracy and precision as well; the process is relatively different from standard protein modeling and is covered in the next sections.

### 1.2. Antibody Structure, Implications for Modeling

**Figure 1:**
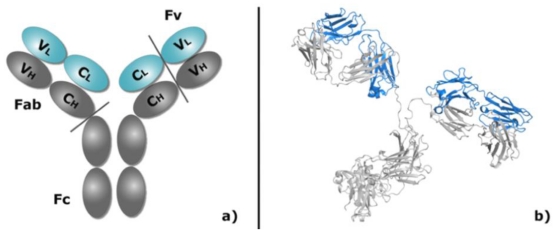
Schematic (**a**) and cartoon (**b**) representation of a full antibody structure. Antigens bind to the tip of the V_H_ and V_L_ domains.

Antibodies are large (~150 kDa), y-shaped molecules containing a so-called Fc region (Fragment, Crystallizable, it binds to various cell receptors and mediates a response of the immune system) and two Fab regions (Fragment, Antigen Binding). The latter are composed by one heavy and one light chain, each with a constant and a variable domain called F_V_ (Figure 1). The F_V_ is the only domain responsible for antigen binding and, therefore, the only one that needs to be considered for docking. It is further subdivided in a framework region, highly conserved in both sequence and conformation, and six highly variable CDR loops (Complementarity Determining Region), three from each chain and often referred to as L1, L2, L3, H1, H2, and H3.

Despite their high sequence variability, five of the six loops (all except H3) can assume just a small repertoire of main-chain conformations, called “canonical structures” [5–7]. These conformations are determined by the length of the loops and by the presence of key residues at specific positions in the antibody sequence. The specific pattern of residues that determines each canonical structure forms a signature that can be recognized in the sequence of an antibody of unknown structure, allowing successful prediction of the canonical structure itself with high accuracy [8,9]. Uncertainties arise in the relatively rare cases when a loop is particularly long and/or does not follow canonical structures. The H3 loop does not appear to adopt canonical structures, instead, and predicting its conformation requires more sophisticated and less accurate approaches.

The framework regions can also be reliably predicted since known structures with high sequence identity are often available. Due to the presence of conserved residues at the interface between the light and heavy chain, the relative geometry of these domains is also well preserved [10]. Correct assembling of the heavy and light chain is nonetheless critical for the accurate orientation of the antigen binding interface and errors may arise in the modeling.

It is important to note that the rules and templates used for modeling are based on structures of antibodies bound to their antigen and are therefore accurate in the context of the bound conformation of an antibody.

### 1.3. Antibody Modeling Based on Canonical Structures, the PIGS Server

PIGS (Prediction of ImmunoGlobulin Structure [11]) is a web-based server for the automatic prediction of antibody structure [12] based on the canonical structure method [13]. The Web Antibody Modelling server, WAM [14], utilizes the same approach but offers less features and is generally less convenient to utilize.

In the canonical structure method, the sequence of each variable domain (V_L_ and V_H_) of the antibody of unknown structure (target) is independently aligned with the corresponding variable domain sequences of all the immunoglobulins of known structure. For this step, standard database searching (e.g., BLAST) [15], and multiple sequence alignment (e.g., Clustalw) [16] programs can be used, but it is important to verify that residues at key structural positions are correctly aligned. The backbone structure of the framework is then modeled using the known structures with highest sequence identity as template. The rationale for this is that, in general, the higher the residue identity in the core of two proteins the more similar the conformation in this region [8] and, hence, the higher the quality of the model. Similarly, the conformation of the CDR loops is predicted using known templates with the same canonical loop conformation and high sequence identity. Different combinations of templates can be used as illustrated below.

- Best heavy and light chains. Use the chains with highest sequence identity as templates. Since they come from different antibodies, the two chains need to be packed together by a least-squares fit of the residues conserved at the interface. This may introduce errors in the relative orientation of the two chains, with adverse consequences for the accurate modeling of the antigen binding site.
- Same canonical structures. Use a template whose CDR loops have the same canonical structures as the target even if a template with higher sequence identity exists for one or both chains. If framework and loops are taken from different templates, then the loops need to be grafted in, possibly introducing errors: the residues adjacent to the loop are superimposed to the framework by a weighted least-square fit of the main chain.
- Same antibody. Use the same antibody as template for both heavy and light chain, even if templates with higher sequence identity exist. This does not require optimization of the relative orientation of the two chains and thus avoids the errors illustrated earlier.
- Same antibody and canonical structures. The template is an antibody with the same canonical structures as the target and it is used to model both framework and the CDR loops. This option does not require optimization of framework orientation nor loop grafting and may offer more accurate results even if templates with higher sequence identity are available for one of the chains. The approach tends to fail, however, if the identity is too low.

The conformation of five of the six CDR loops can be modeled as described but no canonical structure is known for the H3 loop. However, the so-called “torso” region, *i.e.*, the H3 residues closer to the framework, can still be predicted by similarity to antibodies sharing the same torso conformation [17–19]. The “head” region of H3, instead, follows rules of standard protein hairpins and can be predicted by similarity to protein loops (not just antibodies) with high sequence identity, but the result is usually less accurate than for other CDR loops.

The subsequent step consists in the modeling of the side chains conformations. At sites where the parent structure and the model have the same amino acid the conformation of the parent structure is retained. Otherwise, the side chain conformation is copied from antibodies with high sequence similarity or imported from standard rotamer libraries [20]. Finally, the model is refined by a few cycles of energy minimization to improve the stereochemistry, especially in those regions where segments of structures coming from different immunoglobulins have been joined, but not to significantly refine the models.

### 1.4. Antibody Modeling by Rosetta Antibody

Rosetta Antibody [21] is a homology modeling program to predict antibody F_V_ structures. It uses a simple energy function to simultaneously optimize the CDR loop backbone dihedral angles, the relative orientation of the light and heavy chains and the side chain conformations. The program can be downloaded and run on local computers or modeling requests can be submitted to a web server [22,23]. Rosetta Antibody first identifies the antibody templates with highest sequence identity for each framework and CDR loops; the loop templates are then grafted onto the framework and the full F_V_ is assembled. This crude model is used as input for a second stage: a multi-start, Monte-Carlo-plus-minimization algorithm that generates two thousand candidate structures. H3 loop conformations are generated by assembling small peptide fragments [24] and sidechains are finally optimized via rotamer packing and energy minimization [25]. The CDR backbone torsion angles and relative orientation of light and heavy framework are also perturbed and minimized with a pseudo-energy function that includes van der Waals energy, orientation-dependent hydrogen bonding [26], implicit Gaussian salvation [27], side chain rotamer propensities [28] and a low-weighted distance-dependent dielectric electrostatic energy [29]. In the end, a scoring function is used to discriminate the 10 best antibody models that are offered as standard result.

### 1.5. Other Procedures for Antibody Modeling

Methods based on canonical structures are generally very effective but somehow limited by the lack of structural templates for a few loop conformations. Other methods model the CDR loops using templates selected by sequence identity (to other Abs or proteins in general) rather than by the presence of key residues as in the canonical structures method [30–32]. Alternative approaches have been used to model CDR loops with *ab initio* methods based on physicochemical principles [33–38], which have the advantage of not requiring any template and can thus be applied even if no suitable canonical structure is found. Their major limitation is that, due to our poor comprehension of the physicochemical principles governing protein structures, the pseudo-energy functions used to evaluate the different conformations often fail to distinguish a correct prediction. Another limitation is that *ab initio* methods tend to have higher computational costs than similarity-based approaches. As our understanding of protein structure and energy function increases, the combination of the canonical structure procedure with other more sophisticated computational approaches may offer improvements.

### 1.6. The Docking Calculation

Having chosen or generated the starting structures for antibody and antigen, the molecules are then brought together by the preferred algorithm. Computational docking must face two problems [39]: (1) Finding the correct solution, which is usually achieved by changing the relative position of the partners and repeating the calculation thousands of times; (2) discriminating the correct solution from the inaccurate ones by use of a so-called “scoring function”. Simply put, the scoring function rewards positive interaction between the docking partners (e.g., the formation of a hydrogen bond) and penalizes negative interactions (e.g., steric clashes). The assumption is that the correct biological structure is energetically favored and has, therefore, the lowest energy. Scoring functions, also referred to as pseudo-energy, try to simulate this energy by accounting for biophysical considerations such as hydrophobic and electrostatic interactions, salt bridges, hydrogen bonds, *etc*, but also statistical and empirical considerations such as the degree of conserved residues at the interface.

When searching for the correct binding orientation, the two (or more) molecules are allowed to move and the score is assessed after each step. Minimization protocols only retain conformations with a lower energy (better score) than the previous; other protocols (e.g., Monte-Carlo) may retain conformations with higher energy in an attempt to overcome local energy minima that do not correspond to the global minimum. The movement is stopped after a predefined number of steps or when the score does not improve further. The conformational parameters changed between each step vary in different docking algorithms, which may be divided in three general classes as described below: (i) only the relative position of the docking partner is changed; (ii) the relative position and the sidechain conformations are changed; (iii) the backbone conformation is altered in addition to the above.

In the simplest case, the conformation of the starting structures is not altered at all during the docking process and the scoring function only needs to account for the intermolecular interactions [40,41]. This is called “rigid body docking” and exploits the fact that biological interfaces have highly complementary shapes [42–45]. Needless to say, the approach works best if the starting structures are identical to the bound conformation; although this is not too common in biological complexes, good results can nonetheless be obtained in several cases. Various research groups have used this approach [46–52]; amongst them the program ZDock [53] has achieved good results in the CAPRI experiment [54–57].

RosettaDock [58,59] has a first rigid body phase in which sidechains are removed, but in a second phase they are re-introduced and their orientation is optimized [60,61]. Since the sidechain conformation is dictated mainly by a limited number of allowed torsion angles, the task can be completed with reasonable success and limited computational requirements [51].

Accurately simulating the backbone movements that often happen upon formation of biological complexes remains a daunting task for docking, which has a very high failure rate when molecules undergo significant conformational changes upon binding. The degrees of freedom available to protein backbone, especially in loop regions, make it extremely difficult to sample and effectively score the sheer amount of possible stable conformations. Among the programs that incorporate backbone flexibility in the docking [62–64], HADDOCK [65,66] uses a rigid body phase followed by sidechain optimization to select the best scoring decoys, and then simulates backbone flexibility on a selected number of decoys (200 with the default options) in a final stage. It is not practical to run the final stage for all the thousands of initial decoys because of the high computational requirements, although this problem will become less significant with increase in computing power. Likewise, a recent update to RosettaDock adds the option of backbone minimization to the standard protocol with moderate success [58].

It is not clear which approach should work best when docking antibody-antigen complexes. It is conceivable that *in vivo* antibodies adapt to and are selected against existing antigen conformations, thus it might be tempting to believe that antigens should not experience drastic changes upon antibody binding. Rigid body docking might be best in this case, but first of all it is doubtful that proteins are not subjected to any conformational motion in solution, not even at the sidechain level, and furthermore, there are examples in which antibodies provoke relatively large allosteric effects on the antigen [67]. The issue is slightly different for the antibody, instead: since antibody modeling uses bound conformations as templates, the conformational rearrangements experienced by the antibody upon binding can be ignored. It should be noted, however, that the canonical structures used for antibody modeling describe the backbone but not the sidechain conformations, which are probably best explored during the docking run. In conclusion, if one believes that the antibody model is accurate and that antigen binding loops are relatively rigid, then it should not be necessary to sample antibody backbone flexibility in the docking run. This assumption appears reasonable for the five CDR loops following canonical structural rules but it might fail for models of the H3 loop, which may be slightly inaccurate and/or might indeed be flexible in the biological context. Conversely, docking methods that vary the CDR loops’ conformation might introduce deviations from the canonical structure and decrease the accuracy.

Although it is impossible to draw general rules, using rigid body approaches for the backbone but sampling different sidechain conformations might be a reasonable compromise. It might also prove useful to allow backbone movement for the H3 loop (and others when they do not follow canonical structures) while allowing only sidechain optimization of the remaining antigen binding loops. This behavior can also be approximated by generating multiple antibody models, presumably differing mainly in the H3 conformation, and using all of them as starting structures to be docked without backbone optimization, either as an ensemble [68] or serially. Besides requiring more computational time, this approach exacerbates the problem of providing a reliable scoring function: Errors are generated both by the inability to correctly assess intermolecular interactions and also by intramolecular differences amongst the various starting conformations.

### 1.7. Exploiting the Peculiarities of Antibodies to Simplify the Docking Search

Antibodies have a number of features that can be exploited to improve, speed up and simplify the docking search, much like the existence of canonical structures simplifies CDR loop modeling. The recently introduced SNUGDOCK [69,70], for instance, is geared towards antibodies and builds upon the RosettaDock protocol by adding simultaneous optimization of the antibody-antigen position, CDR loops conformation and heavy and light chain relative position.

More generally, since we know that Abs interact with antigens through their antigen binding loops, there is no need to search for possible intermolecular contacts in the rest of the molecule. Typically, the antibody is initially positioned with its CDR loops facing the antigen and it is not allowed to deviate from this general orientation for the entire docking process. This not only increases the calculation speed but also generates much fewer possible models of the bound complex (decoys), easing the burden of scoring and analyzing the results. Constraints can also be introduced to reward the CDR residues for being at the interface, penalize them if they are not or penalize contacts between the rest of the antibody and the antigen. However, particular care must be exercised when introducing constraints or bonuses affecting the final score, since it is relatively easy to force an inaccurate solution or discard a valid, albeit slightly inaccurate one. For example, residues close to the CDR loops but not formally belonging to them can be at relatively close distance to the antigen, therefore rejecting any solution involving proximity of non CDR residues to the antigen would be inappropriate. Conversely, in the known experimental structures not every CDR residues interact with the antigen and forcing them to do so during docking would be a mistake.

## 2. Results and Discussion

Although an extensive benchmarking of modeling approaches goes beyond the scope of this manuscript, we used computational docking to predict the structures of two antibody-antigen complexes recently determined by X-ray crystallography, with the aim of illustrating the potentials, pitfalls and opportunities of antibody modeling and docking.

Most of the antibodies capable of neutralizing influenza virus bind to the highly variable “globular head” region of hemagglutinin, which covers the viral surface. Due to this variability, however, their efficacy is limited to few viral strains and to the narrow timeframe before the virus changes its sequence to prevent antibody binding (anti-flu seasonal vaccines are usually changed every four years for this reason) [71–74]. Two independent research groups have recently described antibodies against the highly conserved “stalk” region of hemagglutinin [75,76]; remarkably, X-ray structures show that they bind to an almost identical epitope utilizing very similar intermolecular contacts, for instance between aromatic residues of the Ab and a conserved hydrophobic patch on the antigen. Antibodies against the stalk have potentially broad reactivity, since the region is conserved in different viral strains, and the virus is not likely to develop resistance against them because of its inability to mutate that part of the molecule. As a consequence, the stalk is pharmaceutically attractive and generating more antibodies targeted against this very region is a worthwhile effort: any computational strategy capable of rapidly and reliably characterizing the binding properties of such new antibodies would be a valuable research tool.

### 2.1. Modeling Antibodies against Influenza Virus Hemagglutinin

**Figure 2:**
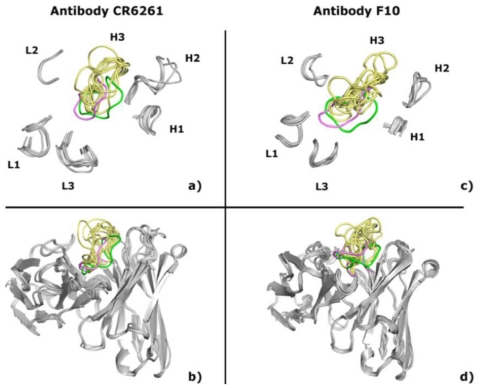
Cartoon representation of antibodies CR6261 (left) and F10 (right). Only the CDR loops are shown at the top, not drawn to scale. The H3 loop is colored green for the X-ray structure, violet for the best PIGS model and yellow for the Rosetta models.

In this work, we used PIGS and the Rosetta Antibody server to predict the structure of antibody F10 (PDB code 3FKU) and CR6261 (PDB code 3GBM) [75,76], results are summarized in Table 1 and cartoon representations of antibodies are shown in Figure 2. The “same antibody and canonical structure” approach (see description in Section 1.3) of the PIGS server is expected to offer the best results, but no viable template with high sequence identity is available. When the “same antibody” method is chosen, PIGS returns accurate results: the RMSD to the experimental structure is about 1 Å for CR6261, either for the whole antibody or individual loops, and 1.3 Å for F10. Choosing the individual chains with highest identity as templates (“best heavy and light chains” approach described above) brings the RMSD of CR6261 to 1.7 Å, still accurate but somehow less precise than before. Curiously, the most problematic loop is H2 and not H3 as it usually happens. Finally, the “same canonical structures” approach yields the worst model, with RMSD of 2.1 Å (2.4 Å in the least accurate loops). In the case of F10, the last two approaches return models with unacceptable steric clashes and highly unusual features that are consequently discarded, while choosing templates with lower sequence identity gives results worse than the “same antibody” option and not further analyzed. Even if no benchmarking is available, the authors’ recommendation to use the “same antibody” approach is in agreement with our general experience and with these particular antibodies.

In contrast to PIGS, Rosetta Antibody does not offer a choice of different modeling methods but, starting from the same templates, returns 10 different models for each target antibody. We refer to them as PIGS or Rosetta1-10 according to the modeling program used and to their relative score, *i.e.*, Rosetta1 is the best scoring model, Rosetta2 the second best scoring and so forth. The best models for CR6261 (e.g., Rosetta2 and Rosetta5 in Table 1) are as accurate as those predicted by PIGS, with RMSD slightly higher but not significantly so; the worst model has RMSD of 1.9 Å (2.5 Å for the H3 loop), still an accurate result. Similar considerations are true for F10, with RMSD of 1.7 Å (2.0 Å for H3) for the best model and 2.6 Å (3.4 Å for H3) for the worst one.

There is no way to assess which of the 10 Rosetta models is best in a blind experiment, when the antibody structure is not known. The model with the best score, thus preferred by the algorithm, is not the most accurate, neither in this example nor in our general experience but, in a worst-case scenario, even selecting the worst generated model would be acceptable. The issue is not overly important as far as docking is concerned, as it will be shown later, and the ensemble of different models might actually represent a conformational flexibility relevant in the biological context.

In summary, all the antibody modeling methods offer satisfactory and, in some cases, surprisingly precise results. Despite the lack of known canonical structures, even the H3 loop conformation can be reliably predicted.

### 2.2. Docking Antibodies against Influenza Virus Hemagglutinin

We used three different programs and approaches to predict the binding interaction of antibodies F10 and CR6261 with the stalk region of influenza hemagglutinin. Several combinations of different starting structures were explored in order to evaluate the docking performance in all possible scenarios. In this manuscript, “model” refers to a starting structure while “decoy” is used to indicate a docking result.

The antigen starting structure was taken from the X-ray structure of the respective complex (bound, PDB codes 3FKU and 3GBM), taken from the X-ray structure of free influenza hemagglutinin (unbound, PDB code 3FK0) or predicted by homology modeling (model) using the I-Tasser web server [77,78]. The structures are rather similar to each other, with pair wise RMSD below 1.6 Å (Table 2).

The antibody starting structures were either taken from the X-ray structure of the complex (bound) or modeled as described above; 10 models were generated by the RosettaAntibody server and one by PIGS for each of F10 and CR6261. We independently docked all the 11 models with the intent of assessing which one is best for docking, but also with the belief that their ensemble represents the biologically relevant dynamic motions available to inherently flexible protein loops.

We initially tested a rigid body only approach with the program ZDock but no acceptable solution (RMSD to the X-ray structure lower than 20 Å) was found in the top 20 scoring decoys when docking either a bound-bound or bound-Rosetta1 combination. Given the negative result in the best conditions for rigid body docking (bound-bound starting structures) the approach was not further evaluated.

RosettaDock was used, instead, to test the performance of a protocol including both rigid body docking and sidechain optimization. Here we assess the accuracy of a decoy by measuring its spatial distance (RMSD) to the available X-ray structure; other indicators, like the number and type of intermolecular contacts, do not alter our considerations and are not described for simplicity. We classify a decoy as “highly accurate” if it has RMSD below or equal to 1 Å, “accurate” if the RMSD is between 1 Å and 5 Å, “acceptable” with RMSD between 5 Å and 10 Å and “poor” if the RMSD is between 10 Å and 20 Å. Unless otherwise stated, all RMSD values indicated in this section are calculated between a docking decoy and the corresponding X-ray structure and reported for the six CDR loops because they are most representative of the binding interface. Complete results are summarized in Table 3 and 4 and detailed results are shown in Supplementary Tables 1 and 2.

The most accurate decoy generated for a bound-bound situation has a RMSD of 2.0 Å for CR6261 and an amazing 0.3 Å for F10. Curiously, the accuracy decreases for F10 when using an unbound or homology modeled antigen structure in combination with a bound antibody structure, as expected, but increases for CR6261 (1.9 Å and 1.1 Å, respectively). It is not evident why starting with a homology modeled antigen should give a result closer to the bound structure than starting with the bound structure itself, but it should be noted that the differences are relatively small and well within the uncertainty implicit in many X-ray structures.

Docking the antibody model generated by PIGS to either a bound, unbound or modeled antigen gives highly accurate or accurate results for F10 (RMSD between 0.4 Å and 1.2 Å) but only acceptable results for CR6261 (RMSD between 5.8 Å and 6.9 Å), even if the two starting models are equally accurate (*i.e.*, more similar to the bound conformation). Also, docking a homology model of hemagglutinin to PIGS F10 is apparently better than docking a bound antigen, but it might be unwise to emphasize the small difference (0.4 Å *versus* 0.7 Å).

Equally good results are obtained when docking the 10 Rosetta models of F10 to bound, unbound or modeled hemagglutinin. The most accurate decoys have RMSD of 0.5 Å while even the worst combinations of starting structures have RMSD below 2.0 Å. Docking Rosetta models of CR6261 with any antigen provides accurate results, as well. The RMSD is higher when using an unbound antigen, which would be understandable if the unbound and bound conformations were significantly different but this is not actually the case. The best results are obtained, somehow unintuitively, with a homology modeled antigen. The Rosetta antibody models of CR6261 are just as accurate as PIGS (similarity to the bound antibody conformation), yet they consistently give more accurate docked decoys. Once more, it seems that the accuracy of the starting structure is not directly correlated to the accuracy of the docking result.

Furthermore, no correlation can be found when comparing the results of the same Rosetta model docked to different starting antigens. Rosetta6 of F10, for instance, gives the best result when docked to a bound antigen (0.6 Å) but the RMSD drops to 1.3 Å and 2.0 Å for unbound and modeled antigen, whereas other models (Rosetta8 and Rosetta9) have 0.5 Å in those cases. Similar considerations are true for CR6261.

It is very difficult, if not impossible, to predict which antibody model should be used for docking in a blind experiment. Different models perform differently when docked to different starting antigens, and the highest scoring antibody model, the one preferred by the Rosetta Antibody algorithm, never gives the best docking results.

As a final test we used the program HADDOCK, which includes a flexible backbone step in the docking protocol, to repeat all the docking calculations performed with RosettaDock. Using the bound conformation as starting structure for both hemagglutinin and antibody gives acceptable decoys with RMSD of 5.6 Å for F10 and poor decoys (RMSD 10.5 Å) for CR6261. The result is considerably less accurate than RosettaDock and things do not improve when an unbound or model antigen structure is docked to a bound conformation of the antibody. This may not be too surprising considering that

HADDOCK changes the backbone conformation during docking, thus forfeiting any benefit of starting from a bound conformer. HADDOCK fails to identify the binding site when docking the PIGS model of F10 (RMSD larger than 30 Å) and offers acceptable solutions for CR6261 (RMSD above 6.8 Å). Docking the Rosetta Antibody models shows a similar trend: the majority of combinations of starting structures fail to identify the correct binding site and the few that do have a RMSD of 5 Å or more to the corresponding X-ray structure. It should be noted that the most accurate decoys have a rather good RMSD for the heavy chain (0.9 Å in the best cases) but the light chain is not predicted as accurately (RMSD of 8.0 Å or more). Visual inspection of these decoys shows that HADDOCK finds the correct position for the heavy chain, which makes most of the contacts to the antigen, but moves the light chain away from the heavy, possibly in an attempt to reduce inter-chain steric clashes. The resulting antibody conformation differs significantly from the experimentally determined one.

It should be noted that we used HADDOCK with the default options available on the web server. Preventing the program from changing the relative position of the heavy and light chain, thus retaining the conformation predicted by the antibody modeling programs, might actually be a better strategy.

### 2.3. Selecting the Most Accurate Solution: the Scoring Problem

So far we have assessed the quality of the predicted complexes by comparing them to the available X-ray structures. This would not be possible, obviously, in a biological research scenario when no experimental information is available (and if it was, computational docking would be pointless). In such a case, the best solutions would have to be chosen according to a scoring function, either external or associated with the program used for docking.

**Figure 3:**
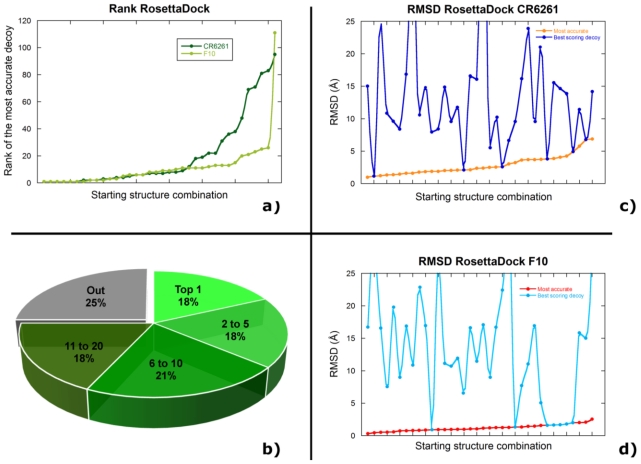
(**a**) Rank of the most accurate decoy (1 is the best scoring decoy, 2 the second best scoring and so forth) for each starting structure combination; (**b**) Rank of the most accurate decoy, presented as a percentage of all the possible starting combinations used; for example, the most accurate decoy is also the top scoring in 18% of the cases. RMSD (in Å) for the most accurate (orange and red) and best scoring (blue and cyan) decoy for CR6261 (**c**) and F10 (**d**). The best scorers are often considerably less accurate then the most accurate decoys, so choosing them as final docking solution would not be ideal. All data refer to the RosettaDock calculations.

The simplest option is to accept the best scoring decoy amongst the thousands generated in a typical calculation. In our Rosetta simulations, the best scorer decoy is also the most accurate (similarity to the X-ray structure) in six out of the 36 possible combinations of starting structures for CR6261, and in seven out of 36 for F10 (supplementary Tables 1 and 2; Figure 3); none of these, however, is the best possible decoy. For example, the top scorer decoy is also the most accurate for the bound-Rosetta10 combination (RMSD 2.1 Å), but the seventh scoring decoy of the bound-Rosetta7 combination has RMSD of 1.0 Å, the best for any CR6261 docking simulation. Even more troublesome is the fact that the best scoring decoy is remarkably wrong for some combinations of starting structures, with RMSD up to 50 Å. The best scorer of Rosetta9 docked to a modeled antigen gives a poor decoy with 16.6 Å RMSD, for instance, but the second best scoring is highly accurate (0.5 Å RMSD).

Although the scoring function is effective in some situations, it is clear that representing the docking solution as an ensemble of the best scoring decoy for each combination of starting structures would not be appropriate (Figure 3c,d). Selecting the best scorer of a single starting combination would be even worse, since we cannot know *a priori* which starting combination offers the best results.

In a slightly different scoring approach, all the decoys are clustered so that similar structures are grouped together; each cluster is then assigned the score of the best scoring decoy within the cluster itself. Finally, the most populated among the five (or whatever is deemed appropriate) best scoring clusters is considered to be the correct docking solution. The assumption is that if the algorithm finds the same good scoring conformation several times, then it might be the best available solution. Although this is correct in some cases, it does not appear to be a valid criterion in most of our calculations: when docking F10 Rosetta4 to a bound antigen, for example, the two most populated and best scoring clusters have 10.8 Å and 23.0 Å RMSD, whereas a scarcely populated cluster outside the top 10 scorers has an accurate 1.1 Å RMSD.

Similar considerations suggest that the presence of a “scoring funnel” is indicative of an accurate computational docking solution. A scoring funnel happens when the algorithm repeatedly finds decoys with similar structure and score significantly better than most others decoys. In practice it is a highly populated cluster whose score is significantly better than any other cluster. However, it is not uncommon for the same starting structures to yield two (or more) different scoring funnels: clearly, not all of them can be correct. Although the authors of Rosetta warn about this problem, it is not uncommon for non-experienced users to rely heavily on the presence of any funnel as an indication of an accurate result.

The program FunHunt [79,80] attempts to distinguish between accurate and inaccurate funnels on the basis of several criteria such as, among others, the number of intermolecular contacts or the average conservation of interface residues. Unfortunately, applying it to some of our hemagglutinin results showed no significant improvement.

### 2.4. Discussion

The importance of antibodies is growing constantly in a number of fields, from the pharmaceutical and biosensor industry to basic research. Their mechanism of action depends, in ultimate analysis, on their atomic interactions with the antigen. Studying and characterizing such interactions is important to understand why antibodies are so effective and to design new molecules with improved properties; identifying the antigen binding site (epitope) is also important for patent claims. Until recently, determining an X-ray structure was the only way to obtain detailed structural information on the antibody-antigen interface. Computational docking, however, is emerging as a fast, attractive and affordable alternative to achieve the same result with reasonable accuracy.

When faced with the task of predicting the structure of an unknown antibody-antigen complex, the first important choice is selecting the starting structures to be docked. The antigen conformation can be obtained from an experimental structure of the free protein or from homology modeling. Although it is difficult to draw general rules and much would depend on the quality of the available structures, both alternatives promise to be sufficient for several interesting targets because more and more experimental structures, to be used directly or as a template for modeling, are available due to an increasing number of structural genomics consortia.

Experimental structures of free antibodies are usually not available, nor would they be very useful since antibodies are known to drastically change conformation upon binding; their starting structures, therefore, need to be predicted in virtually every case. The H3 loop, which shows the most conformational variability in antibody structure, is the most problematic part, but luckily all modeling methods offer accurate and often precise results for the vast majority of antibodies. The already rare events when no reliable antibody template can be found should become even less frequent as the number of available experimental structures increases.

When modeling F10 and CR6261, two antibodies against the stalk of influenza hemagglutinin, it is intriguing that simply copying the conformation of existing templates with the canonical structure method (PIGS) offers better results than optimizing several parameters as Rosetta Antibody does. PIGS is also much faster and results are obtained in a matter of seconds whereas Rosetta Antibody requires about one day on local computers and several days or weeks on the dedicated web server (speed can improve if the queue on the web server diminishes or if larger computer clusters are used locally). Of course we cannot draw general conclusions without benchmarking a large set of models with known experimental structure, but the above appears true in our experience not limited to the two antibodies illustrated here. It is important to note, however, that Rosetta Antibody chooses templates in a way comparable to the “best heavy and light chain” method of PIGS and provides more accurate results than this PIGS option. The optimization run by Rosetta Antibody, in other words, seems to be effective. Both approaches are beaten, however, by using the same antibody as template for both chains, an option offered by PIGS but not Rosetta Antibody. It would be interesting to see the results of the Rosetta Antibody protocol applied to a template chosen with the “same antibody” criteria.

Regardless of the chosen modeling method, the structure of F10 and CR6261 can be predicted within approximately 2 Å of the available experimental structure: a precision comparable to the intrinsic uncertainty of some X-ray structures. Although finding the best possible model might have academic value, even selecting the worst one would provide an accurate prediction. However, we strongly believe that an ensemble of different models, in contrast to a single model, is a more accurate representation of the multiple conformations available to inherently flexible protein loops.

The issue of selecting the very best models is even less significant if they have to be used for docking. Using different starting structures may adversely affect the calculations, as illustrated by the fact that the RMSD of our CR6261 complexes varies between 1.2 Å and 6.9 Å for different starting structures, but there is no apparent correlation between the accuracy of the starting structure (similarity to the bound experimental structure) and the accuracy of the final docking solution. This is particularly striking in the case of F10, where docking results are equally accurate even if the H3 loop is flat in the X-ray structure as in some of the models but faces outward, with a significantly different conformation, in others (Figure 2d). On one hand, this suggests that effectively sampling the intermolecular space is actually more important than choosing a very precise starting model, but on the other hand, it means that it is impossible to predict *a priori* which starting structure would provide the best docking results. One might randomly choose a single starting structure knowing that even the worst result may be satisfactory, but docking multiple conformations (starting structures) might offer an attractive alternative. It must be stressed that we did not perform any comprehensive analysis on a benchmark set [81] and only discuss the example of F10 and CR6261, but the above considerations agree with ours, and others, general experience on the matter.

If choosing different antibody modeling methods has no predictable effect on the calculations, the same is not true for different docking protocols. Although we did not perform exhaustive testing, we have remarkably little success when using a rigid body docking algorithm (ZDock) with F10 and CR6261.

Results are distinctively better when testing a program that optimizes the backbone conformation during docking (HADDOCK), but still fail to predict the correct binding interface for the majority of the cases (RMSD to the experimental structure higher than 20 Å). Although the result might seem disappointing, it should be noted that HADDOCK was designed as a data-driven docking program relying heavily on the use of experimental constraints to drive the calculations and that it does not utilize special features to account for antibody peculiarities. We would expect better results if such features were implemented on the dedicated web server. When HADDOCK finds the correct binding site for F10 or CR6261, however, the accuracy is relatively high for the heavy chain but considerably lower for the light chain. Apparently, the position of the heavy chain is constrained by the antigen but the light chain makes few contacts and is free to move around; optimizing the backbone conformation results in a widening of the gap between the antibody heavy and light chain, possibly in an attempt to relieve steric clashes. Better results might be obtained by locking the relative orientation of the two chains so that they do not deviate from the predicted antibody structure, either through inter-chain constraints or considering the two chains as a single, rigid molecule as RosettaDock does.

RosettaDock optimizes the sidechain but not backbone conformations during docking and provides highly accurate or accurate predictions of the complex between F10 and influenza hemagglutinin with every combination of starting structures. This is less true for CR6261, but even the least accurate predictions (RMSD around 6 Å) correctly identify the general binding site. We had excellent results with RosettaDock when studying antibody-antigen complexes unrelated to influenza, as well.

It should be noted that here we describe the results of a so-called “local search”, in which the antibody is manually positioned around the correct epitope at the beginning of the docking procedure and then only a local, but still relatively large, region around the epitope is explored. This obviously requires some information about the binding site, in absence of which a “global search” around the whole molecule is required. Such a global search requires a much larger number of decoys and, consequently, higher computational resources, but in our experience the final result is comparable to a local search.

Overall, when performing computational docking it seems useful to simultaneously modify the sidechain conformations of the antigen and antibody so that they can adapt to each other, whereas optimizing the backbone conformation might disrupt the predicted bound conformation for the antibody and deteriorate the accuracy of the results. Flexible docking methods will undoubtedly gain popularity as they improve but at this time it might not be necessary to use them for antibody-antigen complexes.

The fact that antibody-antigen docking can obtain highly accurate and surprisingly precise results (RMSD of 0.4 Å in the best cases) is a testament to the excellent work of the researchers in the field and bodes well for the future. What is still severely missing, however, is the ability to recognize the good solutions amongst the thousands generated in a typical docking run. If no experimental structural information is available (and, of course, docking would be pointless if the experimental result was known), one has to rely on a semi-empirical algorithm called “scoring function”.

In our RosettaDock calculations, the top scoring decoy, thus deemed the most accurate by the algorithm, has an RMSD between 2 Å and 52 Å in the various combinations of starting structures tested (Figure 3c,d). Some RMSD values might have been even larger if a global search was performed. Clearly, selecting a docking solution simply on the basis of the scoring function would be a mistake and using clustering or other strategies described above would be equally wrong. Further analysis of the scores gives reasons to be optimistic for the future, however: one of the top three scoring decoys has the most accurate RMSD in 23 out of the 72 combinations of starting structures that we tested with RosettaDock; the number rises to 36% if the top five scores are considered, 57% for the top 10 and 75% for the top 20 (Figure 3b).

If we may be a little provocative, these numbers should be considered a very good result by those interested in the technical side of docking, but they cannot be acceptable in a biological context. Having a 75% chance of finding an accurate solution in the top 20 scores is a testament to the validity of the scoring function, but having a 25% chance of predicting the wrong epitope can be devastating when formulating a biological hypothesis.

The shortcomings of the scoring function have a further implication. When asked to dock any two molecules, the computer will bring them together and find a binding solution. Since the algorithm cannot assess if such solution is correct, it follows that it cannot predict if the two molecules are actually supposed to bind *in vivo*, either. In other words, docking should be limited to partners shown to bind from experimental evidence.

The long-term objective is clear: improving the reliability of scoring functions; but in the short-term we believe that the best, and very necessary, strategy is to utilize rapidly obtained experimental data to filter out the inaccurate docking decoys. Such experimental data can be incorporated in the docking protocol as constraints or, more simply, applied as a filter at the end.

For instance, an important constraint implicit in antibody-antigen calculations is that the antibody must interact with its antigen binding loops. Another approach that we recently proposed uses NMR epitope mapping to select the docking solutions that best agree with the experimentally determined epitope [82]; the advantage is that extensive and precise information can be rapidly obtained for the entire epitope, the disadvantage is that a large amount of purified antibody is required. Alternatively, escape mutants (genetic mutations that prevent antibody neutralization) can be used to identify residues necessary for antibody binding in a viral antigen; site directed mutagenesis can be performed on a non-viral antigen with the same objective. The main disadvantage is that information is usually obtained on a very limited number of residues, not representative of the entire epitope; another problem is that such mutations are likely to be at the interface, since they are required for binding, but are not necessarily so, given the possibility of allosteric effects. Finally, cross-competition experiments are quick, cost effective and powerful experimental methods that can complement antibody-antigen docking. If an antibody with known experimental structure is available, as it is the case for the stalk of influenza hemagglutinin, then it is possible to conduct a simple ELISA experiment to discover if it prevents binding of other antibodies, thus indicating that they share the same epitope [83,84]. The obvious disadvantage is that the strategy is only viable if an apt X-ray structure is available; other problems arise due to allosteric effects or because the Fc region may cause steric hindrance and prevent binding of other antibodies even if the epitope is different. Using cross-competition data against the known F10 and CR6261 antibodies to validate the computational docking results is the most promising approach in the specific case of antibodies against the stalk of influenza hemagglutinin.

## 3. Experimental Section

### 3.1. Antibody Modeling

Antibodies were modeled with the programs PIGS and Rosetta Antibody, using antibodies not related to influenza virus as templates. The PDB codes of the templates for CR6261 are as follows (sequence identity is indicated in parentheses). PIGS: 1RZF for both heavy (63.93% identity) and light chain (78.07%). Rosetta Antibody: 1RZI for the heavy-chain framework (85.07%) and 1Q1J for the light chain (96.72%); 1Q1J for L1 (91.67), 1Q1J for L2 (100%), and 1Q1J for L3 (same length, no identity); 1RZI forH1 (80%), 1RHH for H2 (76.47%), and 1AP2 for H3 (same length, no identity). The PDB codes of the templates for F10 follow. PIGS: 1RZF for both chains (sequence identity 59.29% for the light chain and 62.6% for the heavy chain). Rosetta Antibody: 1RZG for the heavy-chain framework (71.68%) and 1RZF for the light chain (64.36%); 1BJM for L1 (same length, no identity), 1RZF for L2 (83.33%), and 1RZF for L3 (90.91%); 1RZG forH1 (62.50%), 1RHH for H2 (76.47%), and 1FAI for H3 (same length, no identity).

### 3.2. Docking

Hemagglutinin forms trimers on the viral surface and in the available X-ray structures; only the monomeric unit was used for docking to alleviate the computational load. Similarly, only the F_V_ variable domain of the antibodies was docked. The starting structures were visually oriented with the Ab CDR loops facing hemagglutinin and then separated by 25 Å. Since the docking procedure explores a relative large area around the starting position, very careful initial positioning of the docking partners is not required. When using RosettaDock 2.3 the structures were perturbed with 3-8-8 movements (perturbation along the line of centers, in angstroms-perturbation in the plane perpendicular to the line of centers, in angstroms-rotational perturbation, in degrees) and approximately 5000 decoys were generated for each docking run as previously described [82]. The ZDOCK and HADDOCK calculations were performed on the dedicated web servers with default parameters. In HADDOCK, a single Ab residue was constrained to be at the interface (“active residue” as required) whereas the rest of the CDR loops were indicated as possible interface residues (“passive residue” as indicated by HADDOCK). Clustering analysis of the RosettaDock results was conducted with both a 5 Å and 10 Å cut-off.

### 3.3. RMSD Calculations

Backbone RMSD values, calculated with the program ProFit [85] are shown throughout the manuscript. When comparing antigen structures or complexes, the hemagglutinin structures were superimposed and RMSD values were obtained for the subset of residues indicated in the various cases (e.g., H3 loop). The heavy and light chains were superimposed when comparing free Abs.

## 4. Conclusions

Computational docking of antibody-antigen complexes can today achieve excellent results, certainly better than just a few years ago. A pessimist would state that this is true only for the best cases, but to us this is a clear indication of the progress and potentiality of the technique, which can only improve with algorithms and computing power as well as increase in the number of users. It is just as clear, however, that the computational predictions need to be validated, and possibly driven, by rapidly obtained experimental data. Such data is readily available in the form of cross-competition experiments for antibodies binding to the stalk of influenza hemagglutinin, so we are confident that docking can be a reliable strategy to characterize new antibodies against this very important pharmaceutical target.

It is possible that scoring functions will eventually become sufficiently reliable for docking to be used independently, although we believe that experimental validation will always be necessary. In our mind, there is little doubt, however, that experimentally validated computational docking will become an accepted branch of structural biology in the coming years.

## Figures and Tables

**Table 1:** Backbone RMSD values (in Å) between the modeled antibodies and the corresponding X-ray structure. Rosetta and PIGS models are indicated as discussed in the main text. Lc and Hc indicate the light and heavy chain, respectively. The length, in residues, of the 6 CDR loops is indicated in brackets. All generated models are accurate.

	RMSD (Å) C Only												
CR6261	R1	R2	R3	R4	R5	R6	R7	R8	R9	R10	PIGS same Ab	PIGS same CanSt	PIGS Best HcLc
Hc + Lc	1.2	1.1	1.4	1.3	1.1	1.9	1.5	1.1	1.5	1.6	1.0	2.1	1.7
Lc	1.0	0.9	1.2	1.1	0.9	1.6	1.3	0.9	1.2	1.3	0.9	1.9	1.7
Hc	1.4	1.2	1.6	1.5	1.3	2.2	1.7	1.2	1.7	1.8	1.1	2.5	1.7
CDR (all)	1.5	1.3	1.6	2.0	1.3	2.3	2.8	1.8	1.7	1.9	1.1	2.3	1.8
CDR (Lc)	1.2	1.0	1.3	1.8	1.1	1.8	1.4	1.7	1.4	1.5	1.0	2.0	1.7
CDR (Hc)	1.5	1.3	1.7	1.6	1.4	2.4	1.9	1.3	1.8	2.0	1.2	2.4	1.8
L1 (12)	1.2	1.0	1.3	1.5	1.1	1.9	1.5	1.4	1.4	1.5	1.0	2.0	1.7
L2 (4)	1.2	1.1	1.4	1.4	1.1	1.9	1.5	1.3	1.4	1.6	1.0	2.1	1.7
L3 (8)	1.2	1.1	1.3	1.5	1.1	1.9	1.5	1.3	1.4	1.5	1.0	2.0	1.7
H1 (9)	1.3	1.2	1.5	1.4	1.2	1.9	1.6	1.2	1.5	1.6	1.1	2.1	1.8
H2 (5)	1.2	1.1	1.4	1.3	1.1	1.9	1.5	1.1	1.4	1.6	1.0	2.4	2.1
H3 (9)	1.5	1.3	1.7	1.6	1.3	2.5	1.9	1.3	1.8	2.0	1.1	2.4	1.7
**F10**	**R1**	**R2**	**R3**	**R4**	**R5**	**R6**	**R7**	**R8**	**R9**	**R10**	**PIGS same Ab**	**PIGS same CanSt**	**PIGS Best HcLc**
Hc + Lc	2.1	1.9	1.8	1.7	2.0	1.9	2.0	2.6	2.1	2.3	1.3	-	-
Lc	2.1	1.9	1.7	1.7	2.0	1.9	2.0	2.6	2.1	2.3	1.3	-	-
Hc	2.5	2.1	1.9	1.8	2.3	2.1	2.2	3.1	2.3	2.7	1.2	-	-
CDR (all)	2.5	2.5	2.0	1.9	2.4	2.3	2.3	3.1	2.5	2.8	1.5	-	-
CDR (Lc)	2.1	2.2	1.7	1.6	2.0	1.9	1.9	2.5	2.0	2.3	1.3	-	-
CDR (Hc)	2.6	2.3	2.1	2.0	2.5	2.4	2.4	3.2	2.5	2.9	1.5	-	-
L1 (11)	2.1	2.1	1.7	1.7	2.0	1.9	1.9	2.6	2.1	2.3	1.3	-	-
L2 (4)	2.1	2.0	1.7	1.7	2.0	1.9	1.9	2.6	2.1	2.3	1.3	-	-
L3 (7)	2.1	2.0	1.7	1.6	2.0	1.9	1.9	2.6	2.1	2.3	1.3	-	-
H1 (8)	2.1	1.9	1.8	1.7	2.0	1.9	1.9	2.6	2.1	2.3	1.3	-	-
H2 (4)	2.1	1.9	1.7	1.7	2.0	1.9	1.9	2.6	2.1	2.3	1.3	-	-
H3 (12)	2.7	2.3	2.1	2.0	2.6	2.4	2.4	3.4	2.6	3.0	1.4	-	-

**Table 2:** Backbone RMSD values (in Å) between the various starting structures of hemagglutinin used for docking.

	Bound R6261	Bound F10	Unbound	Model CR6261	Model F10
**Bound CR6261**		0.6	1.0	1.4	0.9
**Bound F10**	0.6		1.1	1.3	1.6
**Unbound**	1.0	1.1		1.0	1.1
**Model CR6261**	1.4	1.3	1.0		0.6
**Model F10**	0.9	1.6	1.1	0.6	

**Table 3:** Backbone RMSD values (in Å) between the predicted decoys and the corresponding X-ray structure for CR6261. RMSD values of the most accurate decoy for any combination of starting structures are shown. The columns indicate the starting structure used for hemagglutinin and each row represents a starting antibody structure indicated as described in the main text. Highly accurate solutions are in green, accurate in yellow and acceptable in orange.

	Bound	Unbound	Model
**R1**	1.9	4.9	2.0
**R2**	3.0	4.2	1.3
**R3**	2.5	3.7	1.4
**R4**	2.4	3.7	2.1
**R5**	2.1	3.2	1.8
**R6**	1.6	3.8	1.5
**R7**	1.0	3.9	1.8
**R8**	2.6	3.6	2.6
**R9**	2.4	3.7	1.2
**R10**	2.1	4.1	1.6
**PIGS**	5.8	6.9	6.8
**Bound**	2.0	1.9	1.1

**Table 4:** Backbone RMSD values (in Å) between the predicted decoys and the corresponding X-ray structure for F10. RMSD values of the most accurate decoy for any combination of starting structures are shown. The columns indicate the starting structure used for hemagglutinin and each row represents a starting antibody structure indicated as described in the main text. Highly accurate solutions are in green, accurate in yellow and acceptable in orange.

	Bound	Unbound	Model
**R1**	0.9	0.8	1.3
**R2**	1.3	1.2	1.6
**R3**	1.0	1.0	1.7
**R4**	0.8	1.8	1.6
**R5**	1.0	1.2	0.9
**R6**	0.6	1.3	2.0
**R7**	1.3	0.9	0.9
**R8**	1.6	0.5	0.8
**R9**	1.0	2.0	0.5
**R10**	0.9	1.4	2.5
**PIGS**	0.7	1.2	0.4
**Bound**	0.3	2.1	1.5
